# Paediatric population neuroimaging and the Generation R Study: the second wave

**DOI:** 10.1007/s10654-017-0319-y

**Published:** 2017-10-24

**Authors:** Tonya White, Ryan L. Muetzel, Hanan El Marroun, Laura M. E. Blanken, Philip Jansen, Koen Bolhuis, Desana Kocevska, Sabine E. Mous, Rosa Mulder, Vincent W. V. Jaddoe, Aad van der Lugt, Frank C. Verhulst, Henning Tiemeier

**Affiliations:** 1 Department of Child and Adolescent Psychiatry/Psychology, Erasmus University Medical Centre, Kp-2869, Postbus 2060, 3000 CB Rotterdam, The Netherlands; 2 Department of Radiology, Erasmus University Medical Centre, Rotterdam, The Netherlands; 3 The Generation R Study Group, Erasmus University Medical Centre, Rotterdam, The Netherlands; 4 Department of Epidemiology, Erasmus University Medical Centre, Rotterdam, The Netherlands; 5 ENCORE Expertise Centre for Neurodevelopmental Disorders, Erasmus University Medical Centre, Rotterdam, The Netherlands; 6 Department of Paediatrics, Erasmus University Medical Centre, Rotterdam, The Netherlands; 7 Department of Clinical Medicine, Faculty of Health and Medical Sciences, University of Copenhagen, Copenhagen, Denmark; 8 Kinder Neuroimaging Centrum Rotterdam (KNICR), Rotterdam, The Netherlands

**Keywords:** Neurodevelopment, Neuroimaging, Brain development, Developmental neuroscience, Behaviour, Autism spectrum disorders, Cognitive development

## Abstract

Paediatric population neuroimaging is an emerging field that falls at the intersection between developmental neuroscience and epidemiology. A key feature of population neuroimaging studies involves large-scale recruitment that is representative of the general population. One successful approach for population neuroimaging is to embed neuroimaging studies within large epidemiological cohorts. The Generation R Study is a large, prospective population-based birth-cohort in which nearly 10,000 pregnant mothers were recruited between 2002 and 2006 with repeated measurements in the children and their parents over time. Magnetic resonance imaging was included in 2009 with the scanning of 1070 6-to-9-year-old children. The second neuroimaging wave was initiated in April 2013 with a total of 4245 visiting the MRI suite and 4087 9-to-11-year-old children being scanned. The sequences included high-resolution structural MRI, 35-direction diffusion weighted imaging, and a 6 min and 2 s resting-state functional MRI scan. The goal of this paper is to provide an overview of the imaging protocol and the overlap between the neuroimaging data and metadata. We conclude by providing a brief overview of results from our first wave of neuroimaging, which highlights a diverse array of questions that can be addressed by merging the fields of developmental neuroscience and epidemiology.

**Electronic supplementary material:**

The online version of this article (doi:10.1007/s10654-017-0319-y) contains supplementary material, which is available to authorized users.

## Introduction

The last decade has seen a dramatic increase in the number of large neuroimaging studies in paediatric populations [1–12]. These studies include both children and adolescents who have been prospectively recruited [1–6, 11], as well as initiatives that combine existing paediatric neuroimaging data into large datasets [7–10]. A number of large neuroimaging studies include children and adolescents with specific diagnoses (i.e., Attention Deficit Hyperactivity Disorder (ADHD) [13, 14] and Autism Spectrum Disorder (ASD) [6, 7]), whereas others focus on typically developing children and adolescents [4, 5, 12], twins [15], or population-based approaches [1–3, 11]. The age-at-inclusion of these studies varies from infancy to adolescence, often extending into early adulthood, depending on the key goals of the study. The study designs also are quite heterogeneous, varying from cross-sectional, longitudinal, to accelerated longitudinal designs. A number of these studies are summarised in Table 1 and taken together, the combined results from these studies are providing an invaluable glimpse into typical and atypical neurodevelopment from prenatal life into early adulthood.

**Table 1:** Overview of several large clinical and population-based neuroimaging studies in children and adolescents

References	Study	Design	Population	sample size	Age range (n)	Number of sites
[7]	ABIDE I	Cross-sectional	Autism	539 ASD	7–64 years	16
				573 TD		
[131]	ABIDE II	Cross-sectional	Autism	487 ASD	5–64 years	17
		Longitudinal	Autism	557 TD		
[132]	brainSCALE	Longitudinal	Twins	120	9,9 years (SD 1.4)	1
					12.9 years (SD 12.9)	
[133]	Brazilian High Risk Cohort	Cross-sectional	Enriched for psychopathology	655	7–15 years	
[11]	Generation R	Longitudinal	Population-based	1070	6-9 years	1
				3992	9-11 years	
				800+	12-14 years	
[134, 135]	GUSTO	Longitudinal	Population-based	120 neonates	40.1 wks (SD 4.46)	1
				235 children	4.5 years (SD 0.08)	
[2]	IMAGEN Study	Longitudinal	Population-based	2223	13–16 years	8
[4, 136]	NIMH (Intramural)	Longitudinal	Typical Development, Twins & Clinical	618 TD	5–25 years	1
				800 + Twins		
				270 ADHD		
				>2000 total		
[12]	NIMH (Extramural)	Longitudinal	Typical Development	464	7 days–18.3 years	6
[3]	Philadelphia Neurodevelopmental Cohort	Cross-sectional	Population-based	1445	8-21 years	1
[5]	PING	Cross-sectional	Typical Development	1493	3–20 years	10
[1]	Saguenay Youth Study	Wave 1—children	Population-based	1029	12–18 years	1
		Wave 2—parents				

In addition to neuroimaging studies of typical and atypical child and adolescent development, the growing field of imaging genetics is pushing the borders of sample size, as large numbers of subjects are crucial to elucidate the genetic underpinnings associated with neurodevelopment and psychopathology. The ENIGMA consortium [16], Rotterdam Study [17], and the neuroimaging components of the UK Biobank [18] and German National Cohort Studies [19] are excellent examples of studies or consortiums that merge neuroimaging and genetics with the goal of understanding the genetic-related neurobiology of psychopathology. Whereas many of these studies contribute to our understanding of the neurobiology and genetics associated with ageing, disease, and psychopathology, only a handful of large neuroimaging studies are equipped to study the role of early environmental factors associated with brain development. Studies evaluating the environmental factors associated with neurodevelopment are found at the intersection between developmental neuroscience and epidemiology [11, 20]. Since some early environmental factors are potentially modifiable, understanding how different environmental factors influence brain development can have ramifications in public health, potentially relating to primary prevention of psychiatric or neurological conditions.

However, there are numerous challenges in understanding the interplay between the environment and brain development. One challenge is that while we have gained tremendous knowledge regarding changes in the structural and functional characteristic of the brain from childhood through adolescence, it is still difficult to quantify exactly what optimal neurodevelopment is. If environmental factors are shown to be related to altered brain characteristics in a specific direction (i.e., increased grey matter), while it is possible to show a deviation from typical development, but it is more difficult without behavioural, cognitive, or social cognitive data to show that this deviation is pathological or alters optimal brain development. Measures such as cognition and behaviour, for example, are much easier to explain, since higher cognitive performance and fewer behavioural problems are considered optimal. Optimal development is less clear from the perspective of brain metrics. For example, we often consider that less grey matter is associated with less optimal development, whereas more grey matter is considered more optimal development. To illustrate, children with child-onset schizophrenia [21], bipolar affective disorder [22], and ADHD [23] have all been shown to have decreases in grey matter. However, children with ASD show an increase in GM volumes early in life [24]. While studies using diffusion tensor imaging (DTI) often consider that higher fractional anisotropy (FA) is optimal, some studies have shown that children with ADHD [25] and PTSD have increased FA compared to controls. Finally, we often consider greater brain connectivity as optimal, and yet studies have shown that the use of the psychedelic drug LSD increases global functional connectivity [26].

Since many studies evaluating the neurobiology of psychopathology utilize cross sectional designs, they do not take into account temporal differences in neurodevelopment. For example, grey matter development peaks at different times during development, depending on specific brain regions, such as primary, secondary, and association cortices [27]. Decreased GM during childhood or adolescence could reflect being on one of two sides of an inverted U shaped curve. Thus, the creation of non-linear ‘brain growth curves’ for structural and functional brain development will be extremely beneficial to help define the different characteristics of optimal brain development. Similar to trajectories of height, which can deviate lower or higher from the typical developmental curve due to hypothyroidism or precocious puberty, respectively, mapping the non-linear and growth trajectories of the brain will be useful for defining optimal neurodevelopment. These neurodevelopmental growth curves will be especially helpful to assess the role of environmental factors associated with brain development.

However, the creation of such growth curves requires prospective, longitudinal, population-based studies with large samples sizes. One such study is the Generation R Study, which is a population-based prospective cohort study from foetal life onward [28, 29]. The participants included mothers with a delivery date between April 2002 and January 2006 and who delivered in the city of Rotterdam, the Netherlands. Nearly 10,000 pregnant mothers agreed to participate in the study. The study is multidisciplinary and a vast array of measures has been collected from mothers, fathers, and their children beginning in prenatal life covering multiple domains of health and development.

In September 2009 we initiated the first wave of neuroimaging within the Generation R Study, with a total of 1070 6-to-9 year-old children who underwent an MRI scan [11] and 1307 who took part in a neuropsychological battery using the NEPSY-II-NL [30]. In April 2013 a dedicated wide-bore General Electric 3 Tesla scanner was installed and the second wave of neuroimaging within the Generation R Study was initiated. The goal of this paper is to describe the study design, behavioural measures, and imaging protocol for the second wave of neuroimaging within the Generation R Study. In addition, we provide a summary of results to-date from our first wave of neuroimaging.

## Study design

### Subjects

The children who were recruited were participants of the Generation R Study, which is a population-based longitudinal cohort study of child health and development based in Rotterdam, the Netherlands. An overview of the Generation R study design and population has been described in detail [28, 29]. In brief, all pregnant women who were living within a well-defined region in Rotterdam (defined by postal codes) with a delivery data between April 2002 and January 2006 were invited to participate. A total of 9778 mothers provided informed consent and were recruited.

Rotterdam is ethnically diverse, with approximately 44% of the population being non-Dutch. Recruitment into Generation R reflects this diversity. Of the 9778 mothers, 58% were Dutch, 9% Surinamese, 9% Turkish, 7% Moroccan, 3% Dutch Antillean, and 3% of Cape Verdian descent [28]. Additional detailed measurements of foetal and postnatal growth and development have been conducted in a randomly selected subgroup of Dutch children (n = 1232; known as the ‘Focus Cohort’) and their parents at 32 weeks gestational age and at the postnatal ages of 1.5, 6, 14, 24, 36 and 48 months. These additional evaluations on this subgroup were conducted in a Generation R dedicated research centre. From the age of 5 years onwards, all willing children and their parents with the Generation R Study have had regular visits to a dedicated research centre that includes advanced imaging facilities.

**Fig. 1:**
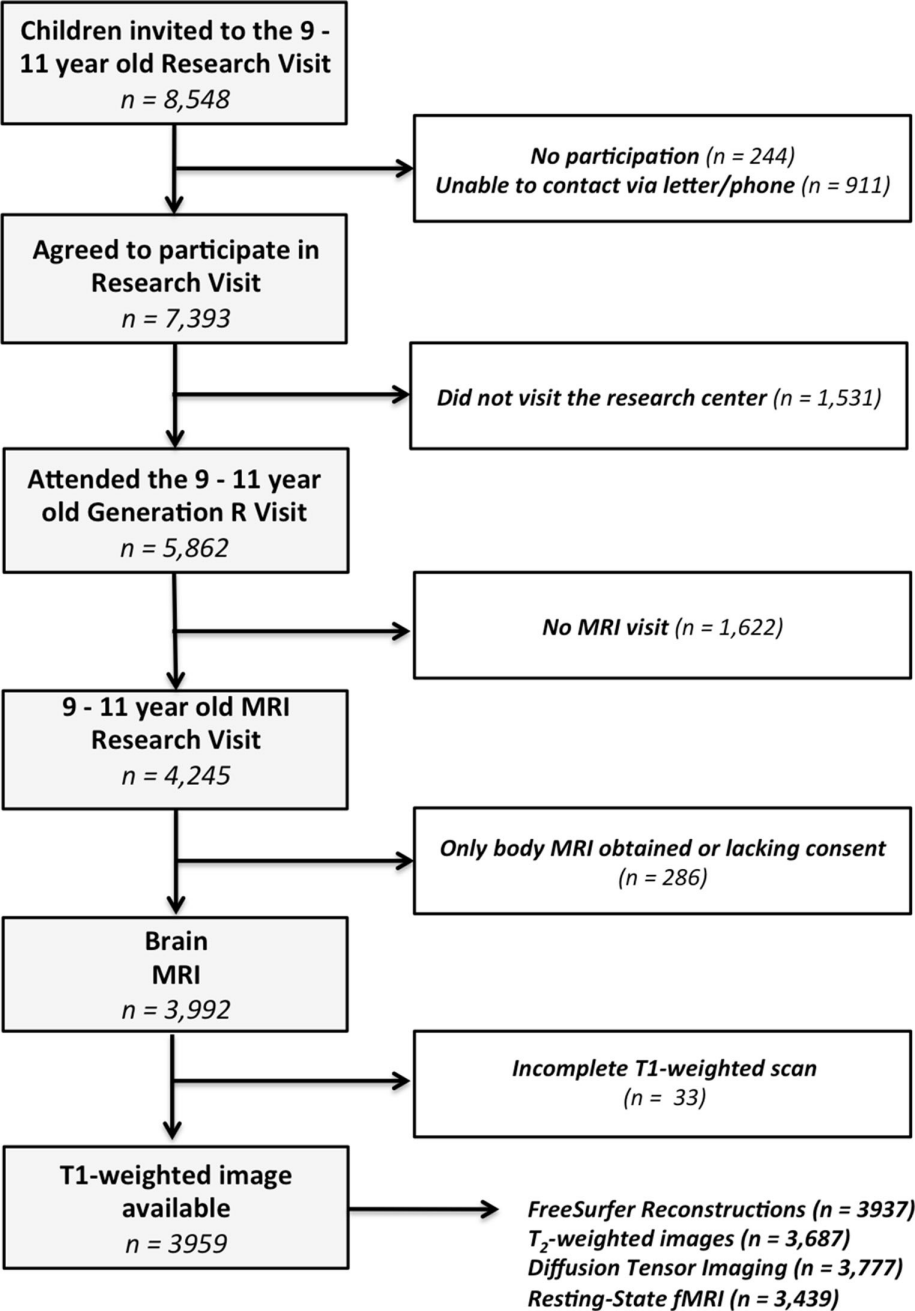
Flowchart of inclusion for the second neuroimaging wave of the Generation R Study

**Fig. 2:**
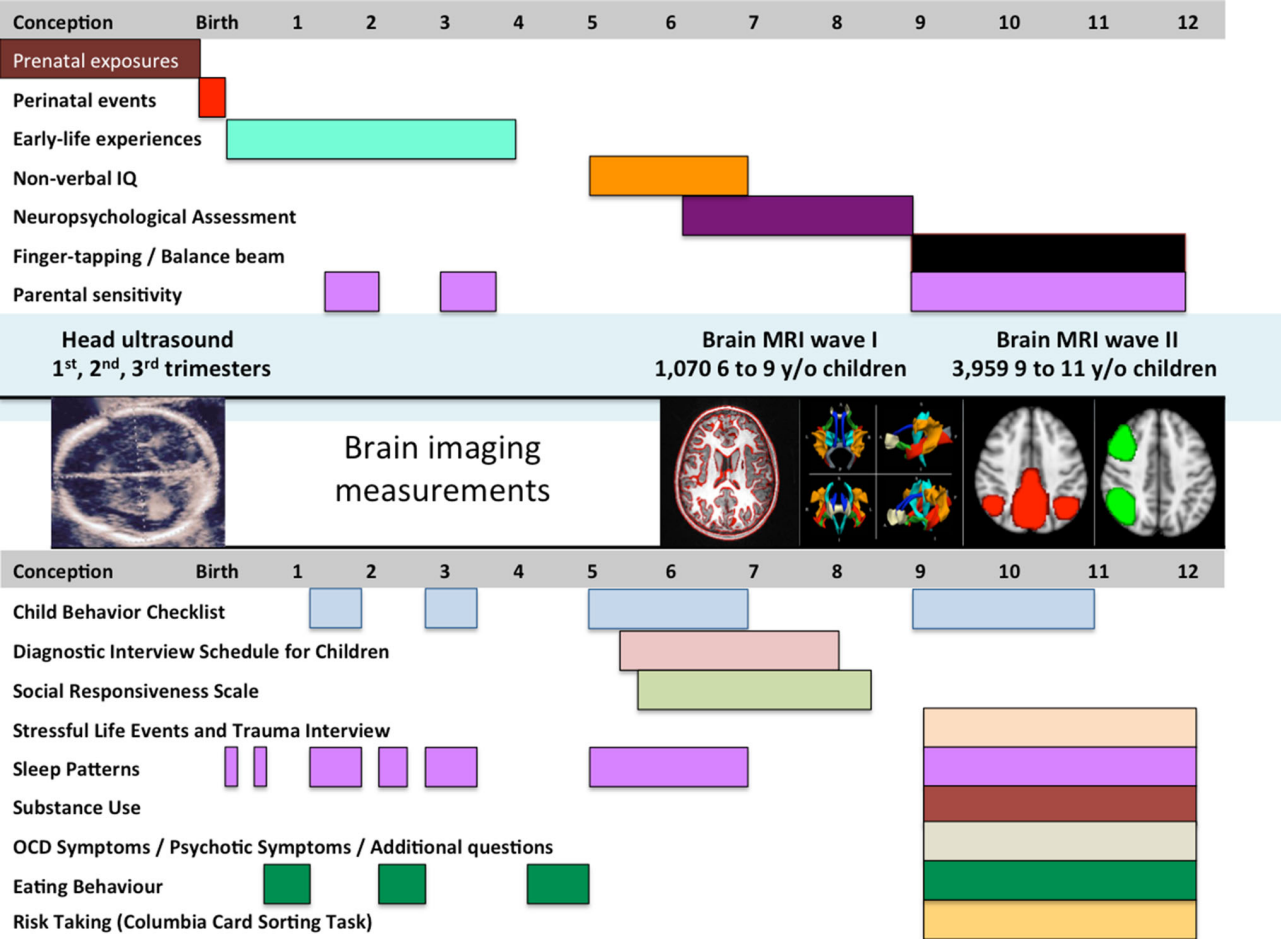
A time-line overview of the major behavioural, cognitive, and neuroimaging data collected within the Generation R Study

The second wave of neuroimaging started in March 2013 with a total of 4245 children visiting the MRI Centre and 4087 children received a brain MRI scan, of which 3992 fulfilled the Dutch laws of parental consent for research and of these 3959 children completed a complete T_1_-weighted sequence, 3687 received a T_2_ scan, 3777 received a DTI, and 3439 received a resting-state-fMRI scan. A total of 3937 scans were successfully reconstructed using FreeSurfer [31]. See Fig. 1 for a flowchart of the recruitment of subjects for the neuroimaging component of the Generation R Study and Table 2 for a description of the demographic characteristics of the study sample. An overview of a subset of key measures collected at different time points within the behavioural and imaging groups of the Generation R Study are shown in Fig. 2.

**Table 2:** Descriptive characteristics of the study population

	n	Descriptive information (mean ± SD or %)
*Maternal characteristics*		
Age at intake (in years)	3992	31.1 ± 4.9
Mean IQ score	3598	97.3 ± 14.8
Educational level at 5 years (%)		
Primary	111	2.8%
Secondary	1244	31.2%
Higher	2087	52.3%
Missing	550	13.8%
Ethnicity		
Dutch	2226	55.8%
Non-Dutch Western	325	8.1%
Non-Dutch Non-Western	1355	33.9%
Missing	86	2.2%
Alcohol use (%)		
Never drank in pregnancy	1347	33.7%
Drank until pregnancy was known	463	11.6%
Continued to drink in pregnancy occasionally	1167	29.2%
Continued to drink in pregnancy frequently	304	7.6%
Missing	711	17.8%
Smoking habits (%)		
Never smoked in pregnancy	2643	66.3%
Smoked until pregnancy was known	303	7.6%
Continued to smoke in pregnancy	475	11.9%
Missing	566	14.2%
*Paternal characteristics*		
Age at intake	3992	33.7 ± 4.9
Educational level at 5 years (%)		
Primary	152	3.8%
Secondary	1120	28.1%
Higher	1884	47.2%
Missing	836	20.9%
Ethnicity		
Dutch	2250	56.4%
Non-Dutch Western	231	5.8%
Non-Dutch Non-Western	1305	32.7%
Missing	206	5.2%
*Child Characteristics*		
Gender		
Boys	1975	49.5%
Girls	2017	50.5%
Gestational age at birth (weeks)	3964	39.8 ± 1.9
Birth weight (grams)	3987	3415 ± 571
Non-verbal IQ at age 5 years	3443	102.5 ± 14.9

Frequent continued alcohol use is defined as ‘1 or more glasses of alcohol per week in at least two trimesters’

**Fig. 3:**
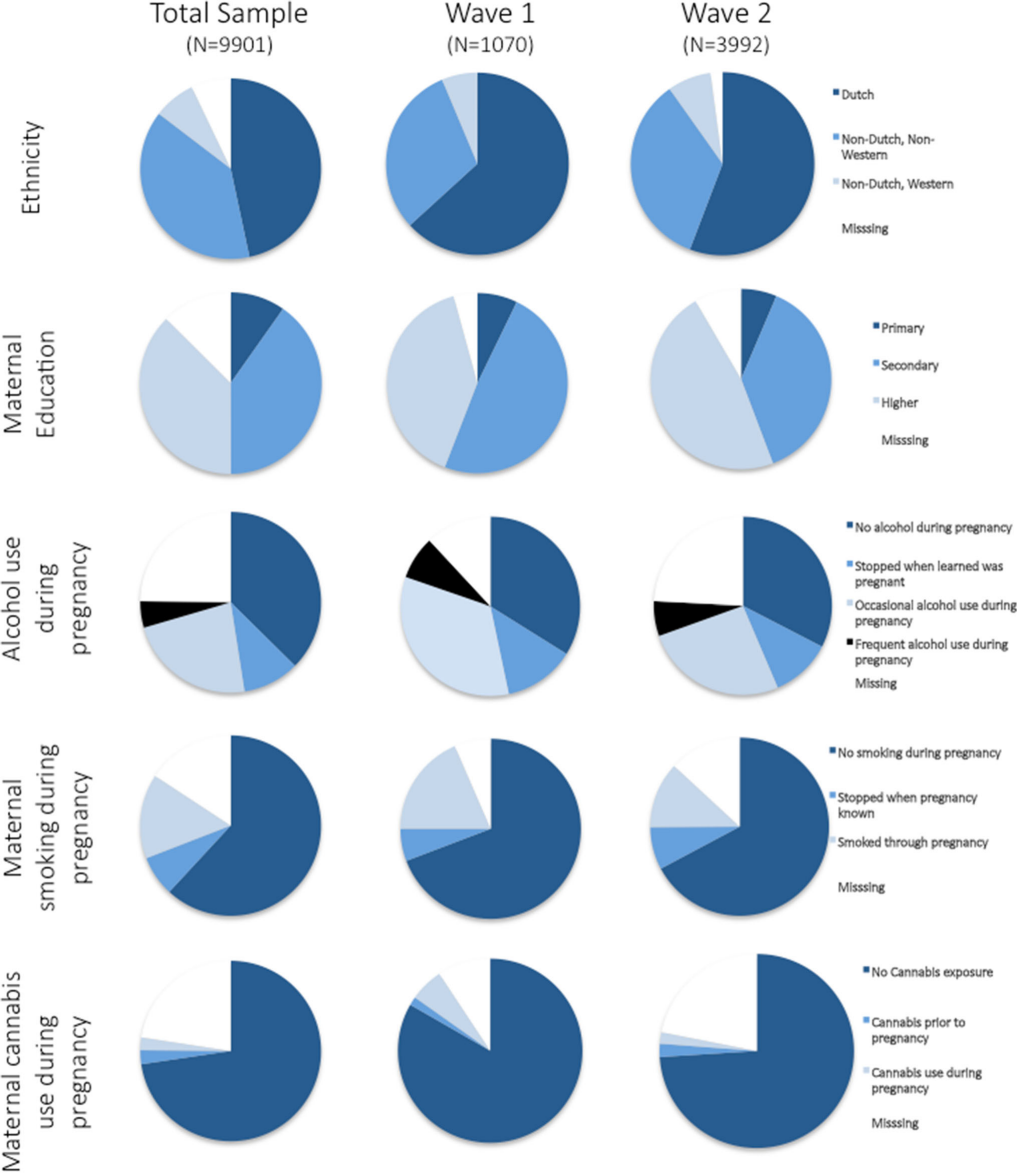
Pie charts reflecting differences in the demographic and pregnancy exposures for the Total Generation R Cohort and for the neuroimaging waves 1 and 2

To assess how well the participant characteristics of the neuroimaging sample reflects the original Generation R Study sample; we performed a non-response analysis using the demographic information at the time of the initial recruitment into the Generation R Study. The mothers involved in the neuroimaging component of the study tended to be on average 2 years older at intake (31.1 vs. 29.1 years of age: t = 18.8, *p* < 0.0001); be earlier on in their pregnancy (14.8 vs. 15.6 weeks pregnant: t = 8.1, *p* < 0.0001); and have children with higher birth weights (3415 vs. 3364 g; t = 4.2, *p* < 0.0001). The families of children participating in the second wave of the neuroimaging study were more likely to be Dutch (*χ* ^2^ = 380.7, *p* < 0.0001), have higher income (*χ* ^2^ = 180.9, *p* < 0.0001), have a higher maternal educational level (*χ* ^2^ = 342.9, *p* < 0.0001), less alcohol use during pregnancy, although those who drank alcohol during pregnancy tended to more frequent alcohol use (*χ* ^2^ = 110.1, *p* < 0.0001), and the mothers were less likely to smoke cigarettes during pregnancy (*χ* ^2^ = 105.9, *p* < 0.0001). Cannabis use by mothers during pregnancy was also slightly greater in those who did not participate in the second MRI neuroimaging wave at 2.5 versus 1.9% (*χ* ^2^ = 9.1, *p* < 0.03). Figure 3 displays pie charts of the frequency of specific demographic factors, showing the considerable overlap between the original recruitment and subsequent imaging waves.

### Magnetic resonance facility

**Fig. 4:**
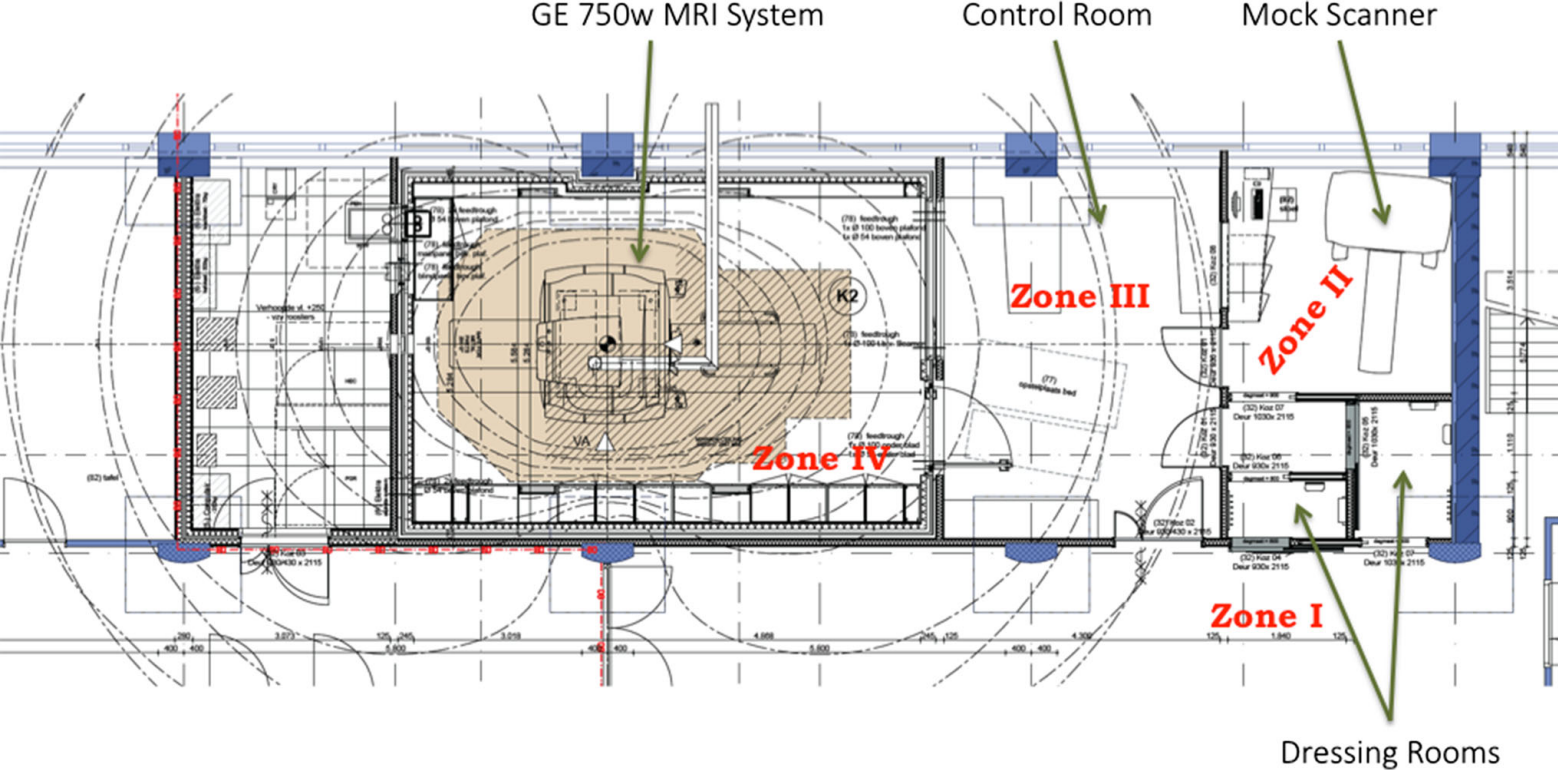
Blueprint of the MRI Suite that was designed to both Optimize Participant Flow and Adhere to the Safety Requirements Set Forth by the American College of Radiology

In early 2012, approval was obtained for the installation of a dedicated research MRI system that would allow large scale MR acquisition from children in the Generation R Study. Plans were made to remodel an area in the Sophia Children’s Hospital to place the scanner. This provided a unique opportunity to carefully evaluate and make changes to the blueprints outlining the remodelling of the paediatric-imaging suite. It was our goal to assure both optimal safety and participant flow. Two researchers (TW and AvdL) carefully reviewed the American College of Radiology Guidance Document for Safe MR Practices [32, 33], and worked with the architects to design the facility along the lines of this document. This included the specifications of Zones I through IV as described in the American College of Radiology Safe MR document [32, 33]. A copy of the final blueprint of the MR suite is shown in Fig. 4.

### Participant flow

Prior to coming to the MR visit, the families had an initial telephone screening where they were asked about any metal that they or their children were wearing or had in their bodies. They were told that the children should wear comfortable clothing (i.e., sweats) without any metal for their visit. Once they arrived at the research centre, the parent was asked in the waiting area (Zone I) to fill in a comprehensive form regarding potential metal in their or their children’s bodies (i.e., pacemakers, past surgery’s, etc.). If the parent was planning on being in the MR room during the scan, they were also asked to fill in one of the forms for themselves. These forms were reviewed for safety and discussed with the families. If there were any questions regarding metal in the body, the children were not scanned until the safety issue was resolved. They then entered one of two dressing rooms that could be locked from both sides. Participants and their parents were then asked to remove any metal objects and leave their valuables, including cell phones and other computer equipment, in one of the two locked dressing rooms. The participants and their parent were asked upon leaving the dressing room if they had removed all metal. They then entered a small hall, which opened into the mock scanner area (Zone II). From the mock scanner room, the participants could walk through a door to the control room (Zone III). The children and their parent were once again asked if they had any metal on them and were asked to check their pockets, hair, or neck for jewellery. At that point, they were led into the magnet room (Zone IV).

### Protection of human subjects

The study was approved by the medical ethics committee (Medisch Ethische Toetsing Commissie) at the Erasmus University Medical Centre. All children included in this report had procedures performed in accordance to the World Medical Association Declaration of Helsinki [34], which was also supported by the ethical principles defined in the Belmont Report [35].

### Respect for persons

The neuroimaging component of the Generation R Study was approved by the Erasmus Medical Centre Institutional Review Board (Medisch Ethische Toetsing Commissie). Prior to participation, informed consent was obtained from both parents when possible, or from the primary parent in the case where one parent was either dead or had no legal relationship with the child. Consent included explaining any potential harms and benefits to the parents and the participation in the study. This was also explained to the children using language at their developmental level. Since children are considered a vulnerable population who warrant greater protection [35], during the neuroimaging portion of the study, we implemented a process of obtaining verbal assent from the child at three different time points. This was performed so that the children could easily stop the study if they desired.

Our protocol included showing the children a card with six faces, the expressions on the faces ranged from very happy to very sad. The children were to rate by pointing to one of the six faces whether they were scared or not scared, or whether they were happy about the procedure or sad [11]. The children were asked prior to the mock scanner, after the mock scanner, and after the real scanner and the children were told that if at any time that if they pointed to the sad face on the card, it meant that they did not want to participate and we would stop. Also, there was a possibility for the children to choose to stop during the MRI scan. Children were given an emergency button; if they wanted to stop because they were scared/anxious they were able to squeeze a ball linked to an alarm system in the control room and the technician would immediately stop the scan and get the child out of the scanner. The most common reason that the children squeezed the emergency button was to use the bathroom. Finally, the use of the practice scanner is child friendly, allowing the children to experience the scanner environment, and to opt out if they so desire, before actually entering the actual MRI scanner [36].

### Beneficence

Neuroimaging at 3 Tesla on a standard clinical MRI scanner is considered less than minimal risk. The greatest risk to human subjects involves the presence of ferrous metal objects in the vicinity of the static magnetic field. Researchers who worked in the MRI scanner area underwent an intensive training course to become certified to work in the MRI setting. Furthermore, we performed thorough screening for contraindications in all children and their accompanying parent. This screening took place outside of the MRI suite in Zone I. The children changed into comfortable clothing in one of the changing rooms (Zone II) and we again asked about contraindications to scanning and asked them whether they were wearing or had any metal objects on them.

We implemented a three-step protocol to evaluate the MRI scans for incidental findings. First, every structural scan was examined immediately following acquisition by the MR technician, operator, or physician who was operating the scanner. Second, a small group of PhD and medical students underwent systematic incidental findings training from a neuroradiologist (AvdL) and were required to identify specific findings from a training set of 50 scans. Once trained, the PhD students rated the MRI scans. If abnormalities were identified on the scans during either the first or second step of the protocol, the neuroradiologist then reviewed the scans. Potentially clinically relevant findings were discussed with the neuroradiologist and the paediatric neurologist. If the findings were thought to be clinically relevant, the parents and family practice physicians were informed and the child was referred to the outpatient clinic for follow-up. While minor incidental findings were present in approximately 1/3 of the children, less than 0.5% of children had an incidental finding on MRI that resulted in a clinical referral [37].

### Justice

The principle of justice considers that both the benefits of research, as well as the burdens, should be distributed among society. Since the Generation R Study was initiated as a population-based birth cohort, those invited to participate included all pregnant women and their partners who lived within the city of Rotterdam. Thus, while the population was restricted to women who were pregnant, there were no other exclusion criteria and efforts were made to match inclusion to the ethnically diverse population of Rotterdam. Exclusion for the neuroimaging component of the study was based only on whether the child had contraindications to enter the MRI scanner (surgical procedures with placement of ferrous metal objects, claustrophobia, etc.).

## Behavioural assessments

### Child Behaviour Checklist (CBCL) (9–11 years of age)

Behaviour problems were assessed using the CBCL for ages 6–18, which a reliable and valid measure for behavioural problems [38]. The CBCL is widely used internationally and it has been found to be generalizable across 23 societies [39]. The CBCL was completed by both the primary and secondary caregiver, who in the majority of cases was the biological mother (95%) and father (98%) respectively. The caregivers rated behaviour problems of the child in the previous 6 months on 113 items using a three-point Likert scale (0 = not true, 1 = somewhat true, 2 = very true). Families received the questionnaires, including the CBCL, prior to their visit to the MRI centre. In our sample we have included 3534 children with both MRI and mother-reported CBCL; and 2602 children with both MRI and father-reported CBCL. In Table 3 we show mother and father report of mean scores on the various sub-scales of the CBCL within the imaging sample, including the percentages falling into the borderline-clinical and clinical ranges using empirically-defined cut-offs [40]. The CBCL version for 6–18 years was collected when the children were between 9 and 11 years of age, however, the CBCL version for 1½ to 5 year old children was collected at three prior time points; namely at 18 months, 3 years, and 6 years of age [29].

**Table 3:** Mother reported child behaviour checklist metrics in 3534 9-to-11 year old children who participated in the 2nd neuroimaging wave: mean scores, proportions with borderline-clinical and clinical problems

	Mean (SD)		Percentage borderline		Percentage clinical	
	Mother report	Father report	Mother report	Father report	Mother report	Father report
Anxious/depressed	2.12 (2.59)	1.95 (2.44)	9.80	8.53	2.07	2.46
Withdrawn/depressed	1.11 (1.61)	1.13 (1.61)	8.38	7.92	2.60	2.54
Somatic complaints	1.48 (1.98)	1.31 (1.72)	7.64	11.07	2.57	2.69
Social problems	1.65 (2.17)	1.71 (2.08)	9.39	9.76	2.66	2.50
Thought problems	1.60 (2.15)	1.57 (1.97)	8.32	7.61	2.74	2.84
Attention problems	3.23 (3.14)	3.25 (3.02)	7.02	9.42	2.35	3.19
Rule-breaking behaviour	0.99 (1.46)	1.07 (1.47)	13.02	7.07	3.59	3.38
Aggressive behaviour	2.80 (3.60)	2.68 (3.52)	7.44	7.07	2.01	2.19
Internalizing problems	4.72 (4.94)	4.40 (3.52)	16.81	18.75	9.14	11.22
Externalizing problems	3.80 (4.70)	3.75 (4.62)	16.21	16.22	10.81	10.49
Total problems	17.39 (15.35)	16.84 (14.61)	16.84	16.03	9.17	9.57

### Diagnostic Interview Schedule for Young Children (DISC-YC) (5–8 years of age)

The Diagnostic Interview Schedule for Children-young child version (DISC-YC) was administered in subsample of the Generation R Study that was enriched for psychopathology [41]. The DISC-YC is a highly structured DSM-IV-based interview administered to caregivers of children aged 3–8 years. Six trained interviewers (including bilingual interviewers) administered the computer-assisted DISC-YC that determines the presence of disorders for a timeframe of 3 months, or 1-year for dysthymia and conduct disorder, by applying algorithms provided by the developer. The current study reports on “all children meeting the DSM-IV symptom criteria” including all children displaying the minimum number of symptoms needed for diagnosis (Table 4).

**Table 4:** DISC-YC confirmed cases in the total 9–11 year old sample of the Generation R Study and in the second neuroimaging wave of the Generation R Study

Diagnosis	Total DISC sample		Neuroimaging sample	
	N cases	DISC^a^	N cases^b^	N controls^c^
*Any disorder*	406	1176	218	460
Anxiety disorders				
Any anxiety disorder	193	1176	101	577
Social phobia	39	1176	22	656
Separation anxiety	23	1175	14	664
Specific phobia	142	1158	71	607
Generalized anxiety disorder	23	1175	9	669
Obsessive–compulsive disorder	11	1175	3	675
Post-traumatic stress disorder	3	1176	1	677
*Mood disorders*				
Any mood disorder	13	1169	7	668
Major depressive episode	4	1172	3	673
Dysthymia	9	1170	4	672
*Behavioural disorders*				
Any behavioural disorder	218	1088	103	526
Any ADHD	207	1169	98	579
ADHD-inattention	67	1170	39	607
ADHD-hyperactive	69	1174	28	619
ADHD-combined	71	1175	31	644
Oppositional defiant disorder	11	1175	108	576
Conduct disorder	13	1039	9	601
*Miscellaneous*				
Nocturnal enuresis	105	1173	56	621
Diurnal enuresis	22	1173	10	667
Encopresis	20	1174	12	666
Tourette’s disorder	2	1174	2	676

Total DISC Sample: Diagnostic Interview Schedule for Children (DISC) Sample’ refers to all children within the Generation R Study who received a DISC, irrespective of whether they participated in the neuroimaging component. Children who were screen positive on the CBCL (n = 1080) and a random selection of screen-negative children (n = 330) were invited to participate in a DISC interview. Thus, the ‘n’ for those who agreed to participate wtih the DISC is 1176

Neuroimaging Sample: This heading describes those within the neuroimaging cohort who had a positive diagnosis (cases) and those who have no DISC diagnosis (controls)

^a^In the column ‘DISC,’ the n for each subtest varied slightly due to periodic inability to collect each subtest of the DISC

^b^ were identified both from the screen-negatives and screen-positives

^c^Confirmed as having no DSM-IV diagnosis based on the DISC-YC

Diagnoses are described without consideration of the impairment measurement score

The target sample within the Generation R Study included children between the ages of 5–8 years who scored in the top 15% of the CBCL-1.5/5 total problems score and the top 2% on the CBCL-1.5/5 syndrome scale scores (screen positives, N = 1080). In addition, a random selection of children scoring below the cut-off points (screen-negatives, N = 330) also received the DISC-YC. Of the 1308 children that were reached, 1176 responded and received a DISC-YC. Of these children, 678 underwent MRI scanning at 9 years of age. In Table 4 the DISC-YC diagnoses (including both screen-positives and screen-negatives) for the 9-to-11 year-old neuroimaging sample of the Generation R Study are shown. In addition, the number in the overall sample of screen-negatives, those who did not undergo diagnostic interviewing are shown.

### Stressful life events and trauma interview (9–11 years of age)

A structured interview was performed by trained PhD students to obtain information on stressful and traumatic events experienced by the child. The interview took place during the 9–11 year old visit to the research centre and was performed only with the primary caretaker, which was generally the mother. The content of the structured interview was primarily adapted from items used in the ‘Stressful Life Events Questionnaire’ developed for the ‘Tracking Adolescents’ Individual Lives Survey’ (TRAILS) study [42]. The interview included a total of 24 questions including topics such as moving, changing schools, death of family, friends, or a pet, unemployment in the parents, parental conflicts, and whether the child has experienced emotional, physical, or sexual abuse. In situations where the caregiver answers ‘yes,’ they were then asked the age of the child when the situation occurred and to what extent it currently influences the child (1 = no influence, 4 = considerable influence). A total of 5587 interviews were conducted, for which 3916 MRI data are also available. Of these, 3755 were conducted with the mother, 143 with the father, and 18 with other caregivers.

### Autistic symptoms (5–7 years of age)

When the children were approximately 6 years of age, their mothers filled out the Social Responsiveness Scale (SRS), which is a questionnaire of autistic traits for children between 4 and 18 years of age [43, 44]. The SRS represents the parent’s observation of the child’s social behaviour during the past 6 months. Each item is scored from 0 (‘never true’) to 3 (‘almost always true’). Higher scores indicate more autistic symptoms. In the SRS both DSM-5 symptom criterion domains for autism spectrum disorder are covered: social communication/interaction and restricted/repetitive patterns of behaviour, interests, or activities. The data collected within the Generation R Study included an abbreviated version of the SRS with a total of 18 items, which has been shown to correlate highly with the full SRS version [45]. The SRS was excluded if over 25% of the questions were missing; otherwise a weighted total score was calculated based on the number of non-missing items. We evaluated a sample of 3857 children aged 4-18 who took part in the Social Spectrum Study in the Netherlands, the correlation between total scores derived from the 18 item SRS short-form and the complete SRS was 0.95 (*p* < 0.001) [46]. The correlation between total scores derived by the SRS short-form and the complete SRS in Missouri Twin Study [47] was 0.93 in monozygotic male twins (n = 98) and 0.94 in dizygotic male twins (n = 134). In a sample of 2719 children from the Interactive Autism Network’s [48] the corresponding correlation was 0.99. We have SRS data in a total of 2983 children with wave 2 neuroimaging data (Table 5).

**Table 5:** Autistic spectrum disorder diagnoses and autistic symptoms measured using the Social Responsiveness Scale in the Generation R Study

Autism related measures	Wave 2 neuroimaging n = 3992		
	Total	Boys	Girls
ASD diagnosis (n)	41	32	9
SRS (n)	2983	1481	1502
SRS (mean/SD)	0.22 (0.17)	0.25 (0.27)	0.19 (0.19)

ASD, autism spectrum disorderl; SRS, Social Responsiveness Scale used to measure autistic symptoms in the general population and children with ASD

### Autism spectrum disorders diagnoses

Since the Generation R Study is a large population-based study of child development, and since the incidence of ASD in the general population is estimated to be between 1 and 3%, it was our goal to identify children diagnosed with ASD in the Generation R Study. To accomplish this, medical records were examined for children that scored screen-positive in one or more of several stages of a multifaceted screening procedure. If a potential diagnosis of ASD could be confirmed through the medical records, the child was considered a clinically confirmed case of ASD. In the Netherlands, the general practitioners hold the central medical records, including information on treatment by medical specialists. A diagnosis of ASD is generally based on clinical consensus by a specialized multidisciplinary team. The diagnostic workup typically involves an extensive developmental case history obtained from parents, as well as school information and repeated observations of the child. To obtain diagnostic information from the family practice physicians, we sent letters to family practice physicians for children that were screen positive for ASD. Screen positive for ASD was based on one of three sources of information. First, all children were formally screened with the SRS. The authors of the scale recommend cut-offs for screening in population-based settings, consistent with short-form SRS weighted scores of 1.078 for boys and 1.000 for girls [49]. In addition, to rule out false negatives, children that scored in the top 15% the CBCL-1.5-5-total score underwent a more specific screening using the Social Communication Questionnaire (SCQ), a 40-item parent-reported screening instrument for ASD [50]. Scores of 15 or above on the SCQ were considered screen-positive [50]. Further, psychiatric diagnoses and treatment were routinely assessed at all contact moments between ages 6–9 (centre visits and questionnaires). All medical records were reviewed by LB, and for questionable cases, there was a consensus meeting with LB, FV, and TW. The number of children with an ASD diagnosis within the Generation R Study, and within the 9-year neuroimaging wave is shown in Table 5.

### Sleep patterns

Sleep patterns (i.e. sleep duration, timing, sleep hygiene etc.) [51] and sleep problems (validated paediatric sleep problems scale derived from the CBCL) were assessed in 7914 children at 2 and 6 months, and at 1.5, 2, 3, 6 and 9 years of age. Of these children, 3867 also underwent MRI scanning. Objective sleep measures using actigraphy are currently being collected in a subsample of approximately 1500 children. In addition, when the children were between 8.5 and 12.5 years of age an adapted version of the Obstructive Sleep Apnea (OSA) questionnaire was obtained in 3881 children [52], of which 2669 children also have MRI data.

### Additional questionnaires covering social, behavioural and emotional domains (9–11 years of age)

Other measures of behavioural characteristics or problems that were collected through questionnaires include, amongst others, prosocial, conduct problems, hyperactivity, and peer problem scales from the Strengths and Difficulties Questionnaire [53], obsessive–compulsive symptoms [54], eating behaviours [54–58] and disorders [59, 60], interpersonal callousness [61], empathy [62], friendship quality [63], self-esteem [64], and use of substances, including smoking, alcohol, and drug use. In addition, measures of school performance, leisure activities, gambling habits, gaming, social media, and television use were collected. We have also assessed internalizing and externalizing behaviour problems through child self-report using the Brief Problem Monitor [65] and specific questions on thought problems from the Youth Self Report [66].

### Additional measures in the focus cohort

Additional detailed measurements of foetal and postnatal growth and development were conducted in a randomly selected subgroup of Dutch children (n = 1232) and their parents. At 30 weeks gestational age the parents underwent an interview to obtain their current and past psychopathology, as well as their parents. At 6 weeks and 14 months of age children received the Touwen test of motor development [67]. The children also had a brain ultrasound through the anterior fontanel at 6 weeks of age [68]. At 14 months of age, attachment was measured using the Ainsworth Strange Situation Procedure [69]. Saliva was sampled at 14 months to measure diurnal rhythm, as well as the stress reaction to the venepuncture. The parents and child were observed and rated during the venepuncture. An electrocardiogram to assess heart rate variability was performed at 14 months. A large battery of tests were performed when the children were 36 months, including the impossible puzzle, gift delay, snack delay, go/no go, peekaboo, Do/Don’t task, emotional facial recognition, stranger approach, bubble blowing, jumping spider, and the puppet game [69–74]. While the Focus Cohort included 1232 children, the sample size and the overlap with imaging differed for each of the above tasks, as not all children and their parents participated in each task.

## Parental measures and family function

### Parental sensitivity (9–11 years of age)

Parental sensitivity towards the child is defined as prompt and appropriate responsiveness towards the child’s signals [75]. Such sensitivity is related to the attachment of the child to the parent [76] and is thought to be important for socio-emotional as well as cognitive development [77]. To measure parental sensitivity, the caretaker and child were asked to draw two complicated figures on an ‘Etch-a-Sketch’ drawing tool within 6 min, with each using only one of two buttons that are needed to draw the figure. The task is virtually impossible and requires a high level of interaction. The scene was videotaped and the timing and adequacy of the caregiver’s instruction and responsiveness to the child is coded [78, 79]. Coding of the data is on-going and we expect an overlap of over 3600 children who also underwent MRI scanning.

### Other parental measures (9–11 years of age)

In addition to measuring the behavioural characteristics of the child, we also obtained information on the behavioural characteristics of the parents and family functioning. From the mother we obtained information on general psychiatric problems using 26 items from the Brief Symptom Inventory (BSI) [80], which covers the depression, anxiety, interpersonal sensitivity, and hostility scales. These same four scales of the BSI were also measured in mothers when the children were 2, 6, and 36 months and the full 53-item BSI was collected when the mothers were at 20 weeks gestational age. Maternal autistic traits when the children were between 9 and 11 years of age was obtained using both the Autism Spectrum Quotient (AQ-Short) [81, 82] and the revised version of the ‘Reading the Mind in the Eyes’ task [83]. General psychiatric problems and autistic symptoms in the father were obtained using an abbreviated form of the BSI and the AQ-Short, respectively. Family function was measured using the Family Assessment Device General Functioning Subscale [84] and family regularity using the Stability of Activities in the Family Environment-Revised (SAFE-R), adapted version [85].

Multiple measures were collected during pregnancy and birth, including blood for biomarkers (i.e., fatty acids, folate, vitamin D, thyroid levels, C-reactive protein, etc.) and pre- and perinatal complications. An overview of selected measures of prenatal and perinatal complications is presented in Table 6.

**Table 6:** Pre- and perinatal factors and overlap with neuroimaging data

	Number	Percentage
	Cases/non-cases	
*Prenatal factors*		
In-vitro fertilization	44/3627	1.2
Intrauterine growth restriction^a^	48/3713	1.3
*Perinatal factors*		
Number of twins	104/3992	2.6
Small for gestational age^b^	51/3473	1.5
Low birth weight (< 2500 g)	211/3743	5.3
Preterm birth (< 37 weeks)	231/3701	5.9
*Delivery*		
Spontaneous or minimally assisted vaginal delivery	2341/3392	71.5
Vacuum- or forceps assisted delivery	501/3407	14.7
Elective caesarean section	177/3407	5.2
Emergency caesarean section	280/3407	8.2
Full breech presentation	41/3642	1.1
Partial breech presentation	128/3642	3.5

	Median (range)	Mean
Gestational age	40.14 (26.3–43.4)	39.8
Birth weight	3440 (635–5610)	3414
Apgar score at 1 min	9 (1–10)	8.6
Apgar score at 5 min	10 (2–10)	9.6
Rank of child (1/more than 1: % > 1)	3595/364	9.2

^a^Intrauterine growth restriction is defined by ultrasound growth which is below the 10th percentile in relation to the gestational age

^b^Small for gestational age is defined by a weight below the 10th percentile for gestational age

## Cognitive measures

### Finger-tapping task (9–11 year old data collection)

To measure motor control, motor speed and lateralized coordination, a computerized finger-tapping task was administered. The children were instructed to tap either with their right index finger, left index finger, or both index fingers in an alternating fashion as fast as possible for a period of ten seconds. The children participated in a total of five trials, involving the right index finger, left index finger, both index fingers, right index finger, and ending with the left index finger. Trials began with both a visual queue and an auditory queue. For the visual queue, an animated image of a hand appeared on the right or left side of the screen for the corresponding trial (right or left taps), or the image of the hand appeared on both sides of the screen for the alternating condition. The auditory queue was a high or low pitched tone to indicate the onset or end of the trial, respectively. Measurements included the total number of finger taps within each trial, and an array of inter-tap intervals for each finger tap within each trial. There were a total of 3752 children with both MRI and finger-tapping data.

### Risk-taking (9–11 year old data collection)

Risk taking was measured using the Columbia Card Task (CCT). The CCT involves 32 cards, displayed in four rows of 8 cards each, shown with each card face down [86]. At the beginning of the task, children had 2 Euros (200 points). Gain cards (with a smiley face) add 1 cent to the trial payoff, and loss cards (with a sad face) end the trial and claim the obtained payoff, if encountered. Children could press the ‘quit and save’ button to save the amount earned at any time during each round. In a total of 24 rounds, children could lose a total of their 2 euros or win up to 5 euros. The top of the screen displayed the following information for a given trial: number of hidden loss cards (out of 32), amount of gain per gain card, amount of loss, and current trial number. Because both the gain and the likelihood of experiencing a loss increase with each turned card, turning over more cards is associated with greater outcome variability and is therefore a riskier strategy. The average number of cards turned over across trials was used as an indicator of a participant’s level of risk taking. A total of 3045 children completed both the CCT and wave 2 MRI scanning.

### Gross motor ability (9–11 year old data collection)

Gross motor development, in particular balance ability, was assessed using the Walking Backwards (Rückwärts Balancieren) task from the Body Coordination Test for Children (Körperkoordinationstest für Kinder) [87, 88]. Data is available in 2916 children that underwent MRI scanning (73.7%). During this task, children had to walk backwards on a balance beam of three different widths. Each balance beam was 3 m in length, and 5 cm in height. After a forwards practice trial on the first beam of 6 cm in width, all children walked twice backwards along each balance beam. The difficulty level of the task increased, as the next beams were 4.5 and 3 cm in width, respectively. Outcome variables were the number of correct steps per trial (with a maximum number of 8 steps per trial) and the amount of time needed to take these steps. If the child did not complete the maximum 8 steps for a trial, then scoring ended once the child fell of the balance beam and touched the floor.

### Neuropsychological functioning

Neuropsychological functioning was assessed in 1325 six-to-nine-year old children using the NEPSY-II-NL, a Dutch adaptation of the NEPSY-II [30, 89]. Due to time constraints, a selection of tests from the NEPSY was chosen such that five areas of cognitive ability could be tapped: attention and executive functioning, language, memory and learning, sensorimotor functioning, and visuospatial processing [11]. The battery was administered by trained researchers and took approximately 55 min to administer. Children were randomly assigned to receive one of four orders of task administration. The instructions in the manual of the NEPSY-II-NL were closely adhered to and the researchers administering the battery did not give positive or negative feedback to children based on their performance.

As the NEPSY-II-NL does not provide domain-specific summary scores or a total score, a data reduction technique was used to derive them empirically [90]. In short, a total performance score for the full battery was derived using a principal component analysis (PCA) on the raw data from all test scores from the NEPSY-II and selecting the first unrotated factor score. Next, for each of the five cognitive domains, a principle components analysis (PCA) was performed on the raw data within each of the NEPSY-II test domains, and again the first unrotated factor score was selected as the summary score for each cognitive domain. The NEPSY-II-NL was performed during the first neuroimaging wave and of the 1070 children scanned, a total of 1053 have both neuroimaging and NEPSY-II-NL data. A total of 724 children have both NEPSY-II cognitive domain scores and wave 2 neuroimaging data, including 639 children with both wave 1 and 2 neuroimaging data.

### Social exclusion task (9–11 year old data collection)

Reaction towards social exclusion and ostracism was measured in a social situation task. This task, the Cyberball task [91], is a computerized game in which the participants were told to play catch with two other children and were asked to imagine how it would be to play the game in real life. The other two children however, are virtual players programmed to exclude the participant after the first six ball tosses. The child then experiences a total of 36 ball tosses, where he or she receives the ball only two more times. Following the game, children were asked to fill out a questionnaire with 15 questions to assess whether specific domains, including belonging, self-esteem, control, meaningful existence, have been threatened (adapted from [92]). Moreover, during the Cyberball game, the participants were unknowingly recorded with a webcam. These videos are being coded for (negative) facial expressions. Currently, analysis and coding of the data is on-going and we expect to have over 3900 children with MRI scanning data available. After the task and filling out the questionnaire, children were debriefed and informed that the other players were virtual and that the exclusion of the other ‘players’ during the game was done on purpose.

### Handedness

We used the Edinburg Handedness Inventory (EHI) to determine hand preference [93]. The EHI contains items related to hand use of 10 items, including writing, drawing, throwing, using scissors, tooth brushing, using a knife (without a fork), using a spoon, using a broom (upper hand), striking a match and opening a box (lid). In addition, two items in relation to eyedness (which eye do you use when using only one?) and footedness (which foot do you prefer to kick with?) were also assessed. Scores were provided for the left and the right hand, foot or eye. The items relating to the use of hands are used to calculate a laterality quotient for each subject [93], which is an index that ranges from -1 extreme left-handedness) to +1 (extreme right-handedness). These data are available in all children with imaging data. In addition, handedness has been measured at two earlier points in time, including during the first wave of neuroimaging data collection using the EHI.

## Procedures and measures of brain growth and development

### Head ultrasound (prenatal data collection)

During the first, second, and third trimesters of pregnancy, foetal ultrasound measurements were systematically performed within the Generation R Study. The first measurement was performed at a mean age of 13.5 weeks, the second at a mean age 20.6 weeks, and the third with a mean age of 30.5 weeks [94, 95]. The ultrasound examinations were used to assess foetal growth patterns and included head circumference and biparietal diameter in up to 3 time points, and ventricular and cerebellar size in up to two time points. For the first ultrasound, the crown-to-rump length was used for pregnancy dating until a gestational age of 12 weeks and 5 days, and biparietal diameter (BPD) for pregnancy dating thereafter. The intra-observer and inter-observer reliabilities of fetal biometry in early pregnancy were excellent (all intra-class correlation coefficients greater than 0.99) [95]. The number of participants with overlap between the structural MRI scans with the first, second, and third trimester ultrasound measures are 2369, 3277, and 3358, respectively.

### Mock scanning session

Prior to the actual MRI scanning session, the children participated in a mock scanning session. The mock scanner simulated the most important aspects of the actual scanning session, including the feeling of being within the bore, wearing headphones in which the child can hear the actual gradient sounds, and the ability to watch a forward-projected film via a mirror positioned on the head coil. The practice scanning protocol is very similar to that used by Durston et al. [36]. Following the mock scanner session, the child was shown two pictures of an MRI scan of the brain, one with little movement and one with considerable movement. This was done to help the child visualize that the ‘pictures of the brain become blurred with movement,’ and we found it quite helpful.

### Magnetic resonance equipment

MR images were acquired on a 3 Tesla GE Discovery MR750w MRI System (General Electric, Milwaukee, WI, USA) scanner using an 8-channel head coil. Care was taken so that the children were comfortable in the scanner and soft cushions were used to assist with head immobilization. The children were able to watch a film of their choice during the structural MRI and DTI. The film was then turned off during the resting state functional magnetic resonance imaging sequence (rs-fMRI).

### Scanner hardware and software

The majority of the children were scanned using a standard, receive-only 8-channel head coil on the GE DV24 scanner software platform (n = 3594). During the initial setup of the scanning wave, a small number of children were scanned on the DV23 software version (n = 365) using a 24 channel head/neck coil (n = 241) and the above mentioned 8-channel head coil (n = 124).

### Imaging sequences

The specific scanner sequences for the structural, diffusion weighted, and resting-state functional magnetic resonance images are presented in Table 7. The majority of the high-resolution T_1_-weighted sequences were obtained using a 3D coronal inversion recovery fast spoiled gradient recalled (IR-FSPGR, BRAVO) sequence using ARC acceleration. However, 21 scans at the start of the study were collected using ASSET acceleration and slightly different parameters. The diffusion weighted imaging data was acquired using an axial spin echo, echo planar imaging sequence with 3 b = 0 scans and 35 diffusion weighted images. The resting-state fMRI sequence involved 206 volumes and was acquired using an interleaved axial echo planar imaging sequence. The interleaved acquisition proceeded inferior-to-superior, beginning with odd slices, followed by the even slices (i.e. [1 3 5 … 35 2 4 6 … 36]. The total duration of the resting-state scan was 6 min and 2 s, which we have shown to be long enough to produce stable resting-state networks [96].

**Table 7:** Parameters for the Magnetic Resonance Imaging Sequences used in the 9-to-11 year-old wave of the Generation R Neuroimaging Wave

Sequence	n	TR/TE/TI (ms)	Flip angle (°)	Field of view (mm)	Acquisition matrix	Slice thickness (mm)/Number of slices	In-plane resolution (mm)	R	Bandwidth (kHz)	Fat saturation	Frequency encoding direction	Phase encoding direction
IR-FSPGR	3938	8.77/3.4/600	10	220 × 220	220 × 220	1.0/230	1.0 mm^2^	2	25	`Yes	S/I	R/L
IR-FSPGR	21	7.82/2.13/600	10	220 × 220	220 × 220	1.0/226	1.0 mm^2^	2	25	`Yes	S/I	R/L
3D Turbo SE	3687	1400/130	90	256 × 256	256 × 256	1.0/176	1.0 mm^2^	2	100	`Yes	S/I	P/A
DWI	3777	12,500/72.8		240 × 240	120 × 120	2.0/65	2.0 mm^2^	2	250	Yes	R/L	P/A
RS-fMRI	3439	1760/30	85	230 × 230	64 × 64	4.0/36	3.4 mm^2^	2	250	None	S/I	P/A

IR-FSPGR, Inversion Recovery Fast Spoiled Gradient Recalled, T_1_-weighted image; SE, Spin Echo, the 3D fast/turbo spin echo sequence was collected using the GE 3D-CUBE sequence, T_2_-weighted image; DWI, Diffusion Weighted Image; RS-fMRI, resting-state functional magnetic resonance imaging; TR, Repetition Time; TE, Echo Time; TI, Inversion Time; R, Acceleration Factor (Asset n = 21, ARC n = 3938)

### Assessment of scanner stability

To monitor the stability of the MR system, sequences were regularly collected on two types of phantoms. The resting-state sequence was performed each morning on an agar phantom and this data was automatically run through the fBIRN quality assessment algorithm. At one point during scanning there was a marked deviation in signal from the agar phantom, which required further investigation into the potential for scanner instability. However, it turned out that phantom had gone bad, with the presence of cysts. In addition to the agar phantom, a phantom to measure geometric distortion was also performed once per week.

### Assessment of Image quality

At the time of the MRI acquisition, T_1_ images were rated for image quality using a six-point Likert scale. The quality assessment levels for the scans were: unusable, poor, fair, good, very good, and excellent. The visual inspection measures used to make this assessment included the sharpness of the gray matter and white matter interface on the cortex, the presence of ringing in the image, and whole brain coverage. If the initial T_1_-weighted scan was rated as unusable or poor by the technician running the scanner, the T_1_ sequence was repeated. A repeat scan took place 381 times (9.5%), and was primarily a result of excess movement. Prior to repeating the scan, communication took place between the child and MR technician to make sure that the child was comfortable and to remind the child to remain as still as possible.

**Fig. 5:**
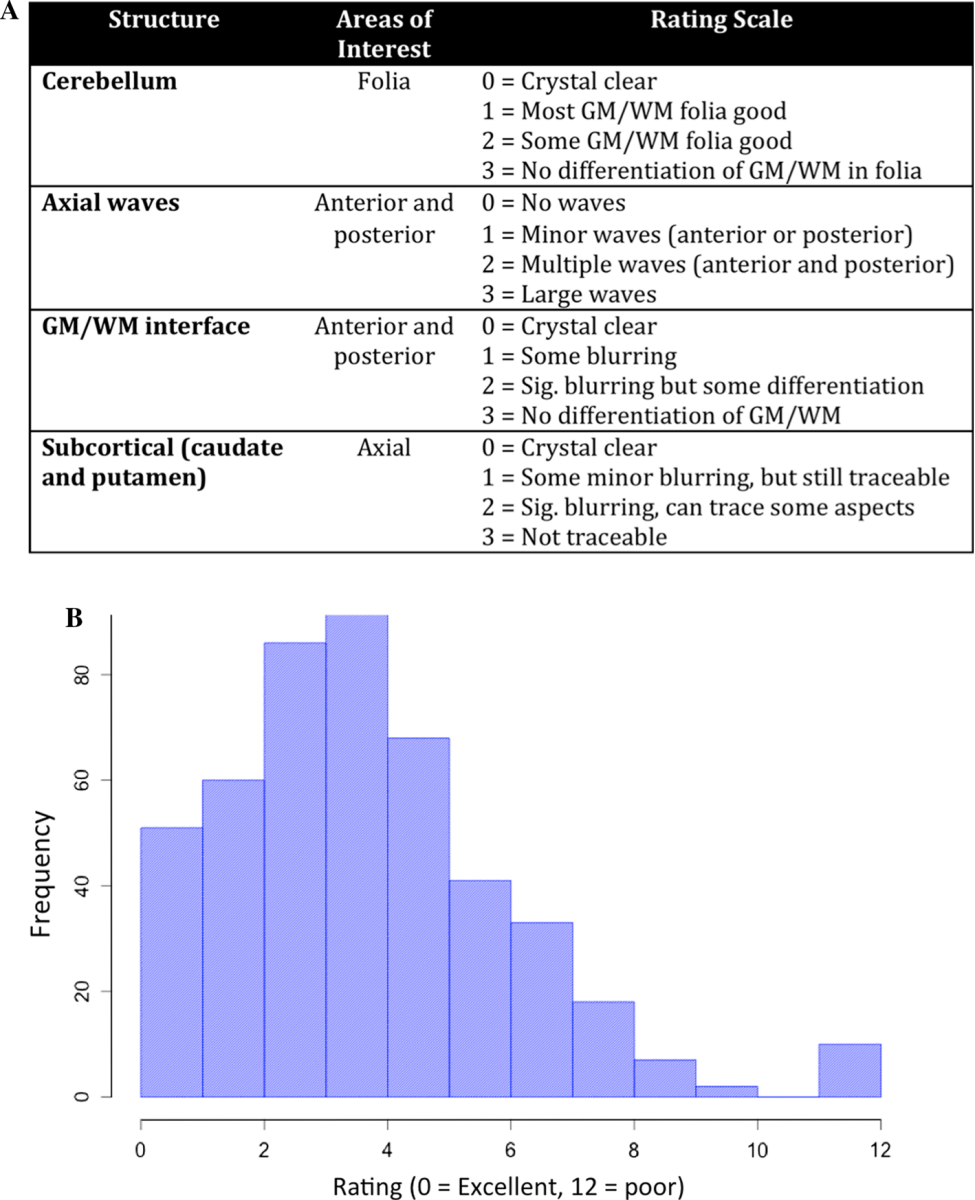
**a** Systematic Quality Assessment Rating Scale for Structural MRI Scans. **b** Distribution of 500 scans rated using the Systematic Quality Assessment Rating Scale for Structural MRI Scans

**Fig. 6:**
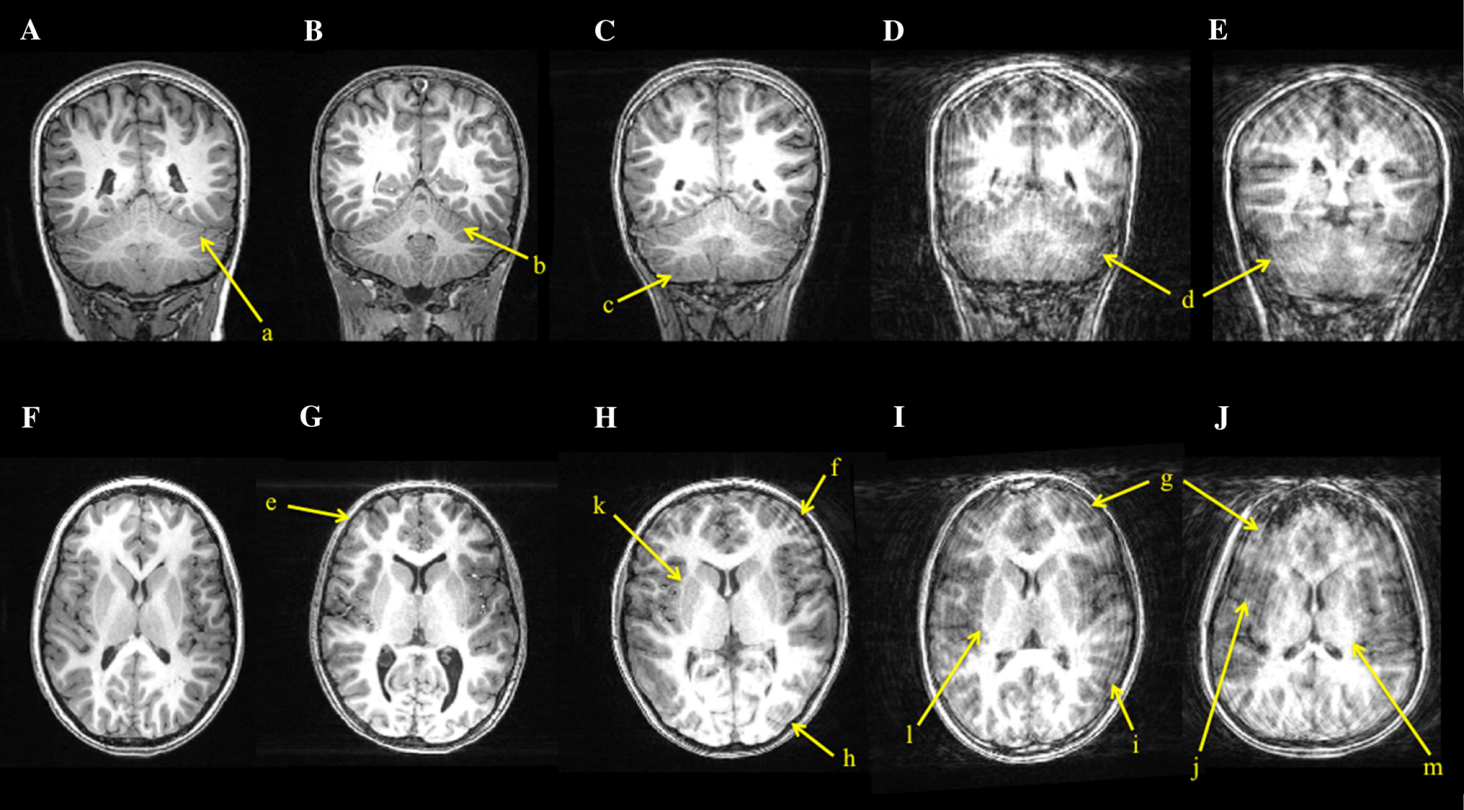
Examples of different quality of structural neuroimaging data using the Systematic Quality Assessment Rating Scale for Structural MRI: **a** crystal clear foliation in the cerebellum, **b** good differentiation in the grey matter/white matter contrast in the cerebellar folia, **c** some blurring of the grey matter/white matter contrast in the cerebellar folia, **d** poor or no differentiation of the grey matter/white matter contrast in the cerebellar folia, **e** no axial waves and good grey matter/white matter contrast, **f** significant waves anterior, **g** large waves or ringing, **h** minor waves posterior, **i** significant blurring of grey matter/white matter contrast, **j** loss of grey matter/white matter contrast, (k) good differentiation of caudate and putamen, (l) minor blurring of the caudate and putamen (m) loss of grey matter/white matter margins of the caudate and putamen rendering it untraceable

In addition to the initial raw T_1_ rating at the scanner, a random sample of 500 scans were systematically rated according to criteria shown in Fig. 5a, with a distribution of these ratings shown in Fig. 5b. The rating was performed systematically using coronal and axial slices and evaluating four different features of the image, demonstrated in Fig. 6. The first feature was rated using a coronal slice cutting through the midline of the cerebellum to examine the details of cerebellar foliation. Our experience is that cerebellar folia are excellent in discriminating between excellent and very good quality images because minor movements of the head can distort the fine details found in the gray/white interface of the foliation. The distortion of the foliation can best be visualized in the superior and inferior regions of the cerebellum. Axial slices were used to evaluate three additional metrics, including axial banding in the anterior and posterior regions, the gray/white matter interface of the cortical rim, and the characteristics of the caudate and putamen. Ratings of image quality of the caudate and putamen tended to differentiate images with fair quality from those that were poor. Each of these four features was rated on a four point Likert scale, with a range from 0 (excellent image quality) to 3 (very poor image quality) and summed for a total score (range zero to twelve). Using this rating approach, intra- and inter-rater reliability were determined using this approach. The intra-rater reliability, measured by ICC was 0.88 and the mean inter-rater reliability between the two scans from rater 1 and rater 2 was 0.72.

There were a total of 88 children who had dental braces at the time of scanning. These children completed only a T_1_-weighted scan that will be used only for analyses involving the cerebellum and occipital lobe. For whole-brain analyses, exclusion of participant based on incidental findings on their MRI scans will involve a two-level approach. First, a total of 26 children with notable incidental findings (tumors, large cysts, agenesis of the corpus callosum, etc.) will always be excluded from these analyses. Second, we will perform sensitivity analyses excluding children with other minor incidental findings (n = 288 for T_1_-weighted image analyses), but which could potentially bias the results (i.e., smaller posterior fossa cysts, enlarged or asymmetric ventricles, etc.).

### Longitudinal Cohort

The number of children included in this report of the second wave who also have been scanned during the first wave of scanning includes 640 children with two T_1_ weighted images; 600 with two DTI scans, and 525 children with two rs-fMRI scans. The mean age in the longitudinal set at wave 1 was 7.6 years (range 6.1-10.6, SD = 0.87), at wave 2 was 10.2 years (range 8.9-11.98, SD = 0.65), and the average time between the two scans was 2.6 years.

## Genetics

Blood for genetic analyses of the children was collected either from cord-blood at birth or from venepuncture at the Generation R research centre. Genotyping was performed using Illumina 610 and 660 K genotyping platforms. More detailed information about the collection and quality control procedure has been described previously [97]. In total, 5732 participants had high quality genotype data available. Of these participants nearly half (n = 2511) participated in the wave 2 MR scanning session. For an overview of the genetic principal components of this sample compared to the HapMap3 founder populations, see Supplementary Figure 1. Based on the principal components, 1462 children of these children were within the range of European ancestry (Hapmap3 CEU).

## Data sharing

There are currently major efforts among many research institutions worldwide to collaborate and/or share neuroimaging and behavioural data to better elucidate the neurobiological underpinnings of both typical and atypical brain development. While the Generation R Study data is not yet openly available, the study is very open to collaboration and shared initiatives. Open data initiatives require considerable infrastructure and resources necessary to assure the protection of human subjects data, to provide the mechanisms for external researchers to understand the variables, to anonymize the data, and to provide seamless data transfer. This is especially true for the Generation R Study, considering the large number of variables and neuroimaging data available. Data sharing or collaborative work within the Generation R Study will fall under the current laws of the Netherlands and European Union. Completely anonymized data can be openly shared with researchers, whereas de-identified data must be shared under the rubric of a data-sharing agreement. In addition, specific data such as photo and film material, and data that could potentially be pieced together to identify specific individuals (i.e., neighbourhood, school, police involvement, etc.), must remain within the Erasmus University Medical Centre. Thus, within a very positive outlook toward data sharing, the future goal of the neuroimaging, behavioural and cognitive component of the Generation R Study is to move toward an open data sharing policy, while assuring the protection and privacy of the children and their families, and adhering to the EU and Dutch laws for data sharing.

## Discussion

Paediatric population neuroimaging is an emerging field that forms an intersection between the disciplines of epidemiology and developmental neuroscience [11, 20]. Developmental neuroscience focuses on the timing and underlying mechanisms associated with brain development and studies span from molecular to gross neuroanatomical levels. Epidemiology focuses on the distribution and determinants of health-related factors or events in specified populations with the goal to improve health [98]. Combining these two definitions, it is the goal of paediatric population neuroscience to improve health through the study and identification of determinants in the population associated with the timing and underlying neurobiology of brain development and deviations in brain development. In some ways, there are specific factors that make population-based neuroimaging studies somewhat different than most hypothesis-driven neuroimaging studies.

One primary goal of population-based neuroimaging studies, similar to epidemiological studies, is to use research criteria to systematically and prospectively collect optimal measures that can be utilized in hypothesis testing. Developmental neuroscience approaches are typically hypothesis driven and designed to test specific underlying mechanisms. As such, developmental neuroscience studies are typically highly selective in the population studied, so as to reduce bias associated with confounding factors, whereas population-based studies have few restrictions on recruitment, but require large numbers to accurately model potential biases. Finally, neuroimaging studies that grow out of developmental neuroscience tend to have much richer cognitive and social cognitive measures, whereas those that grow out of epidemiology tend to have a much richer assortment of early environmental and psychosocial factors that can potentially influence neurodevelopment. Neuroimaging within the Generation R Study falls into the latter, having rich measures of environmental, parent and child health, genetic, epigenetic, and psychosocial factors that date to prenatal life. The advantages of merging developmental neuroscience with epidemiology can be described by highlighting the results from our neuroimaging studies to date.

### Effects of prenatal and early life exposures on downstream brain development

Prenatal life is a period of tremendous growth and development of the brain [99, 100]. The brain differentiates from ectoderm shortly after conception and by the time of birth, usually 40 weeks later, the brain shows the characteristic convolutions found in an adult brain [101]. Neuronal migration begins at approximately 6 weeks gestational age, and continues until approximately 24 weeks [102]. During the third trimester the brain undergoes considerable growth, with the maturation of primary sulci and the formation of secondary sulci [103]. The brain undergoes growth up to approximately 12 years of age and developmental changes in brain structure and function continue well into adulthood [104].

Considering the dramatic global growth that takes place during prenatal life, it is our general hypothesis that influences during this period will likely have global effects on brain development. However, since the brain has regional differences in the rates of brain maturation, global effects could potentially show up, or become ‘unmasked,’ in different areas at different ages. For example, areas that are undergoing the greatest developmental changes may show greater differences, or alternatively, as regions mature, the underlying differences could become unmasked. With considerable data collected during prenatal and early life, the Generation R Study is in a unique position to address questions related to environmental factors in early life and their effect on downstream brain development [105].

Not only is there the opportunity to evaluate downstream brain development with MRI, but also prenatal growth using head ultrasound measures, which were collected during early, middle, and late pregnancy in over 5000 pregnant mothers [28, 106]. Within the first wave of MRI data collection within the Generation R, we performed several studies evaluating whether aberrant prenatal growth due to various exposures persisted into childhood. With the continued development and the inherent plasticity of the early developing brain [107], it would be reasonable to postulate that prenatal differences in growth would be obscured with time. However, such was not the case. Decreases in prenatal growth related to maternal cigarette smoking [106], cannabis use [108], and low maternal folate during pregnancy [109] showed long-term neurodevelopmental differences 6–9 years later [109–112]. These studies have very relevant public health messages that refraining from substance use and acquiring adequate nutrition during prenatal life is crucial for assuring optimal brain health in offspring. Finally, we have also shown that parenting measures during the first 4 years of life, including maternal and paternal sensitivity to their child, was associated with larger total brain and grey matter volumes in school age children [113].

### Psychopathology along a continuum

The introduction of the Diagnostic and Statistical Manual of Mental Disorders, Third Edition (DSM-III) in 1980 was incredibly important for fostering greater precision and reliability in neuropsychiatric research [114]. Based on European approaches of the time, the DSM-III allowed researchers from different institutions around the world to study patients who present with similar clinical phenotypes. Critiques emerged over the DSM-III, including in the field of child and adolescent psychiatry [115]. However, the intensions of the authors of the DSM was for it to represent a ‘best effort,’ rather than being ‘ground truth’ [114], stressing the importance of a thorough understanding of the clinical phenotype. Within this framework, it is not surprising that the conceptualization of psychiatric disorders in research settings has been undergoing a slow, but steady paradigm shift over time. This shift is to evaluate clinical phenotypes not only as categorical (i.e., DSM-based diagnoses), but also within a dimensional framework (i.e., continuum of symptoms within the population) [116].

Within this paradigm shift, large population-based studies, and especially birth cohorts, are extremely well suited to study disorders along a continuum. In birth cohorts, children are recruited before knowing where they will fall on the continuum of behaviour, and barring non-random attrition, these children will reflect of the spectrum found within the general population. Obtaining a valid representation of children with subclinical symptoms, those children who exhibit some symptoms but do not present in a clinical setting [117], can be identified using birth cohort studies. Furthermore, in the case of attrition, the earlier collected data can be used for non-response analyses to provide an indication of potential bias in the sample.

One of the key goals of our work is to study the relationship between psychopathology and developmental neurobiology along the continuum in the population. If the clinical phenotype can be found along a continuum, then it would be reasonable to assume that the neurobiology underlying the clinical phenotype also lies along a continuum. This paradigm shift toward psychopathology along a continuum is also present in the field of genetics, where studies are showing relationships between additive models of genetic risk for psychopathology (polygenic risk scores) and associated clinical symptoms. The confirmation of the nature of these relationships can provide important information regarding the underlying neurobiology of the disorder.

We have applied a three-prong approach that allows for both testing for the relationships within both clinical and subclinical groups and also allows for comparisons with case/control studies [46]. First we test for a linear relationship between the neurobiological variable of interest and the continuous measure of clinical symptoms. Second, we perform a case/control analysis, where we evaluate children who reach a clinical threshold for symptoms (i.e., DSM diagnosis, clinical threshold). Finally, we exclude those participants who score above the clinical threshold for symptoms and assess whether the relationship remains even after the exclusion of those children.

Interestingly, we have used this approach to evaluate cortical morphology and continuous measures of autistic symptoms in children from the general population [46]. A region in the left temporal, the precuneus area demonstrated a linear relationship, where greater autistic symptoms were associated with less gyrification. This finding was significant when children with clinical symptoms of ASD were excluded, providing evidence for a linear relationship across the ASD spectrum. However, the gyrification in the right temporal and frontal region did not show the same negative relationship and had a much small effect estimate when excluding children above the ASD clinical threshold, suggesting that this region may fit a non-linear pattern. The linear versus non-linear relationship between clinical symptoms and the underlying neurobiology would likely involve differences in the interplay between genes (additive vs. threshold effects), which we will pursue in future research. Finally, we have also evaluated attention problems [118], aggression [119], sleep [120], and prosocial behaviour [121] along the continuum and found a linear relationships within specific brain regions.

### Interdisciplinary research

The Generation R Study is an epidemiological prenatal cohort study with the goal to study health and development across multiple disciplines, thus multiple disciplines are involved in the study. These include obstetrics and gynaecology, pulmonology, cardiology, growth and development, dental, ophthalmology, immunology, and endocrinology. Multidisciplinary crosstalk within the Generation R Study provides a unique opportunity to evaluate the interface between developmental neuroscience and other paediatric disciplines, especially regarding environmental exposures, health characteristics, pre- and perinatal complications, and characteristics of early development in association with neurodevelopment.

An example of such collaboration that involves the role of thyroid hormone levels during pregnancy on later cognitive and brain development. The current clinical approach to hypothyroidism is that hypothyroidism during pregnancy should be treated, whereas high thyroid function has no adverse consequences. In collaboration with the Department of Endocrinology, we performed several studies evaluating the role of maternal thyroid and iodine levels during pregnancy. We found evidence of lower IQ in children who experienced lower maternal hypothyroxinemia (subclinical hypothyroidism) during pregnancy [122]. However, while we initially found no differences in brain morphology related to hypothyroxinemia, when the relationship was further tested using quadratic models, it was shown that the model fit an inverted U-shaped curve, with both low and high concentrations of maternal free thyroxin associated with lower IQ and cortical grey matter [123]. Finally, we found no differences in urinary concentrations of iodine in the mother and downstream cognitive differences in their offspring at the age of 6 years [124].

### Typical brain development

With the variations in the patterns of fissures and folds of the brain, the differences in brain shape and size, and alterations of connectivity through experience depending pruning, it is somewhat difficult to exactly define ‘typical’ brain development. But similar to a fingerprint, in spite of each brain being unique, there are characteristics of brain development that are typical, and these involve both form and function. A better understanding of the neurobiology of emerging psychiatric disorders can be obtained by learning more about deviations in these typical elements of neurodevelopment.

**Fig. 7:**
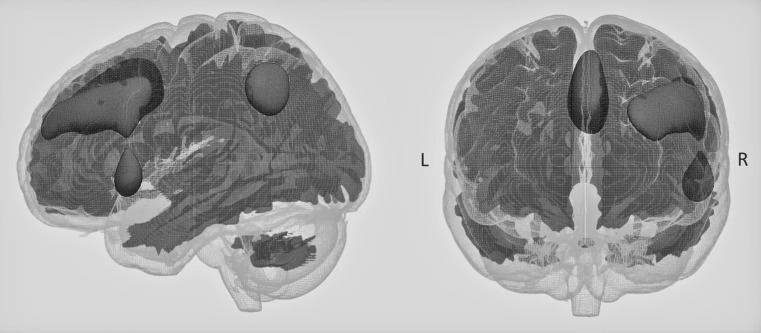
The Right Parietal-Prefrontal Brain Network in 6-to-9 year-old children defined within the Generation R Study. This network showed greater connectivity associated with higher non-verbal IQ

Since psychiatric disorders are often associated with cognitive deficits, we have been interested in the relationship between cognitive function and brain development. Studies in adults have found that connectivity between the parietal and prefrontal lobes are related to general cognitive function, in what is coined as the Parietal-Prefrontal Integration Theory (PFIT). Since the prefrontal cortex has a protracted development into early adulthood, we were interested if the P-FIT theory also held in school age children. Interestingly, we found that non-verbal IQ was related to connectivity between the right parietal and prefrontal regions (Fig. 7), providing evidence for the P-FIT theory in 6–8 year old children [125]. This was further substantiated in structure–function associations observed with diffusion imaging metrics [126]. Fractional anisotropy in the superior longitudinal fasciculus, a large fibre bundle interconnectivity the parietal, frontal and temporal lobes, was also associated with non-verbal IQ [126].

Interestingly, many well-defined resting-state networks observed in adults have also been observed in young children [127, 128]. We showed that not only are these networks present in school-age children, but they are also highly robust [129]. To test the robustness of these networks in children, we performed resampling with replacement to determine those networks that are highly replicable. Specific resting-state networks, including the posterior default mode, sensory, right parietal-prefrontal, and sensorimotor networks were always present in 500 individual resampling analyses. However, other networks, such as the lateral visual, and lateral middle frontal were less robust. Furthermore, we found evidence for age-related increases in network connectivity between the precuneus and lateral frontal networks, and age-related decreases in connectivity between the parietal and the sensory and right frontoparietal networks [129]. These age-related associations were present even within the narrow age band of 6–9 years of age in our first neuroimaging wave. This age range thus represents a period where the brain is undergoing rapid maturation.

### Imaging genetics

Studies investigating genetic determinants of imaging phenotypes are often hampered by relatively high costs of the data and time-consuming data collection. To overcome this problem, large-scale international collaborative efforts have sought to combine multiple, smaller studies and perform meta-analyses, and sometimes mega-analyses using available data. These large sample sizes are needed to study the typically low effect sizes of individual genetic variants on brain structure [16, 130]. As the price for genotyping one individual is decreasing annually, genetic data will become an increasingly more valuable tool in scientific research focused on the development of children.

Large-scale genome wide association studies (GWAS) on behaviour are only starting to uncover the complex genetic construct of behaviour-related traits. The picture that emerges is that many variants of low effect play a role and are common in the general population, additively leading to an increased genetic liability [131, 132]. Population-based imaging studies will play an important role in identifying pathways that explain how genetic liability to specific traits, including schizophrenia and cognitive ability, can lead to a brain that is more likely to develop the trait. Polygenic risk scoring methods, summarizing the additive effect of thousands of common genetic variants, provide a useful method for quantification and subsequent studying of genetic predisposition [133].

### Emerging psychopathology

Studying the neurobiology of emerging psychopathology typically involves either population-based or high-risk studies. Studies of children at-risk include either children who are genetically at-risk or children behaviourally at-risk. However, children who are behaviourally at-risk are already showing behavioural symptoms, and thus have progressed beyond the premorbid state. There remain many unanswered question regarding premorbid neurodevelopmental trajectories of children who later develop severe psychopathology. When in the course of development is there a deviation from the typical developmental trajectories? Are there changes in the brain that can be seen even before the onset of clinical symptoms with these changes become ‘unmasked’ with later neurodevelopment? Or alternatively, does neurodevelopment follow the same pattern of typically developing children; with at some point a deviation in the trajectory at the same time as the illness begins? Since most studies recruit children either with the onset of some symptoms, or after the onset of their disorder, there is little information regarding the neurodevelopmental trajectories leading up to the disorder. Large population-based studies provide an optimal source to obtain neuroimaging data prior to the onset of illness in order to be able to address the pre-morbid status of the brain.

## Conclusion

The neuroimaging component of the Generation R Study has a number of highly unique elements that can address multiple questions within the fields of developmental neuroscience and epidemiology. These include key public health findings stressing the importance of factors associated with optimal brain development; studies evaluating the neurobiology of psychopathology along the continuum; and studies directed at obtaining a better understanding of typical neurodevelopment. The latter is crucial, since it is important to have a firm understand of typical neurodevelopment in order to better understand deviations from typical development. Whereas many neuroimaging studies lack a direct translation from research findings to clinical health, some of the findings in the Generation R Study have immediate important public health messages. For example, we have shown that even in the absence of neural tube defects, low folate can have longstanding effects on the developing brain [110]. In addition, we have shown that smoking during pregnancy results in a relatively widespread decrease in (cortical) grey matter. However, there is evidence that the children of mothers who quit smoking when they learn that they are pregnant do not show the same differences in brain morphology as the smoking group. Thus, for optimal brain health we have shown that both the use of prenatal folate and not smoking during pregnancy can enhance brain development.

Even though in many respects quite unique, the Generation R Study is one of several large neuroimaging studies in paediatric populations. Both the existing studies and the emerging studies will provide crucial information for the development of ‘growth curves’ of optimal brain development, coupled with a better understanding of the factors that can impair the optimal growth and development of the brain. Learning from the current approaches used in genetic studies, the best chance for neuroimaging to have the greatest impact would be utilize the combined strengths of both population-based and developmental neuroscience studies to address important questions surrounding brain health and development.

## Electronic supplementary material

Below is the link to the electronic supplementary material.

Supplementary Figure 1 Principal components plotted for the subsample with available genotype and imaging data collected at 9 years along the HapMap3 populations. African = Hapmap3 YRI, Japanese = Hapmap3 JPT, Chinese = Hapmap3 CHB, European = Hapmap3 CEU. A = First two principal components explaining most of the variation, B = Second and third principal component C = Third and fourth principal component (TIFF 1074 kb)
