# Supplementary figures and images for: Paediatric population neuroimaging and the Generation R Study: the second wave
Source: Eur J Epidemiol. 2017 Oct 24;33(1):99–125. doi: 10.1007/s10654-017-0319-y (PMC5803295; doi:10.1007/s10654-017-0319-y)

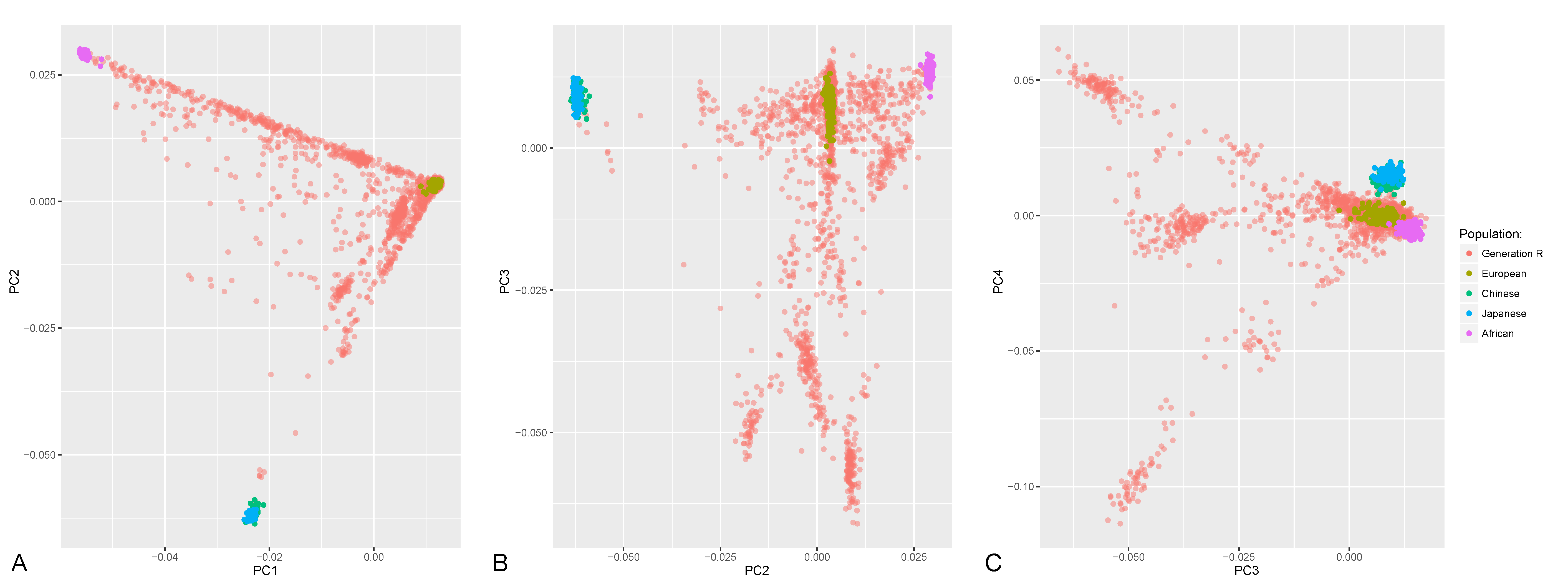

Supplement: Supplementary file 1 — Principal components plotted for the subsample with available genotype and imaging data collected at 9 years along the HapMap3 populations. African = Hapmap3 YRI, Japanese = Hapmap3 JPT, Chinese = Hapmap3 CHB, European = Hapmap3 CEU. A = First two principal components explaining most of the variation, B = Second and third principal component C = Third and fourth principal component (TIFF 1074 kb) [file 10654_2017_319_MOESM1_ESM.tiff]
